# Role of von Willebrand factor (VWF), platelets, and aberrant flow in the initiation of venous thrombosis

**DOI:** 10.1126/sciadv.adr5250

**Published:** 2025-02-05

**Authors:** Laura J. Mereweather, Daniel Harwood, Josefin Ahnström, Joseph van Batenburg-Sherwood, Isabelle I. Salles-Crawley, James T. B. Crawley

**Affiliations:** ^1^ Centre for Haematology, Department of Immunology and Inflammation, Hammersmith Hospital Campus, Imperial College London, London, UK.; ^2^ Department of Bioengineering, White City Campus, Imperial College London, London, UK.; ^3^ Cardiovascular and Genomics Research Institute, School of Health and Medical Sciences, City St George’s University of London, London, UK.

## Abstract

Deep vein thrombosis is a major cause of morbidity and mortality worldwide. However, because of the absence of overt blood vessel damage, how venous thrombosis is actually initiated remains unclear. Using endothelialized fluidic devices, we show that aberrant flow patterns that may occur in venous valve pockets of individuals with common stasis-related risk factors can cause the formation of von Willebrand factor–platelet tangles that are resistant to ADAMTS13 removal. These von Willebrand factor–bound platelets specifically recruit neutrophils in a manner that is dependent on platelet-activated α_IIb_β_3_, neutrophil SLC44A2, and endothelial P-selectin. The interaction of SLC44A2 with activated α_IIb_β_3_ promotes formation of prothrombotic neutrophil extracellular traps. These data provide molecular and cellular insights into the proclivity for venous thrombosis to develop in venous valve pockets and suggest an alternative strategy to protect against the initiation of venous thrombosis.

## Teaser

ADAMTS13-resistant von Willebrand factor–platelet tangles are the nidus for venous thrombosis development.

## INTRODUCTION

Deep vein thrombosis (DVT) is a major cause of morbidity and mortality worldwide. In the United States and Europe combined there are an estimated ~670,000 venous thrombosis-related deaths per year (*1*). Globally, DVT incidence is one to two events per 1000 per year, increasing to 10 events per 1000 per year in individuals >55 years (*2*). DVT risk is increased by surgery/trauma, cancer, and infection, along with several additional lifestyle and genetic modifiers that further influence prothrombotic drivers in venous thrombosis (*3*). Reduced/aberrant flow in the venous valve pockets due to immobilization or sedentary lifestyle is a frequent component, which is highlighted by the “stasis-related risk factors” including surgery, bed rest, pregnancy, plaster cast immobilization, and long-haul travel.

DVT most commonly develops in the deep veins of the legs where the valve pockets represent the nidus for thrombus development (*4*–*11*). Moreover, unlike arterial thrombosis, venous thrombosis involves little or no vessel damage meaning that thrombi develop over an intact endothelium, demonstrating a distinct mode of thrombus initiation. Murine models of venous thrombosis, particularly the inferior vena cava stenosis model, have provided important insights into the initiation of venous thrombosis (*3*). The ability to initiate thrombosis through flow restriction alone and without vessel damage highlights the major influence of reduced/aberrant flow in disease pathophysiology. Despite their limitations, these models, which involve development of thrombi that share many of the hallmarks of human thrombi, have revealed important roles for von Willebrand factor (VWF), platelets, endothelial (rather than platelet) P-selectin, neutrophils, and neutrophil extracellular traps (NETs) in both initiation and progression of thrombus formation (*12*–*15*). We previously demonstrated using a fluidic system that platelets binding to immobilized VWF at arterial shear rates undergo shear-dependent mechanosensitive signaling causing intraplatelet Ca^2+^ release that “primes” but does not fully activate platelets in a classical manner (*16*). This results in activation of the platelet integrin, α_IIb_β_3_, but does not cause appreciable degranulation or second messenger release (*16*). VWF-bound/primed platelets can then recruit neutrophils in a manner that is dependent on activated α_IIb_β_3_ (with a minor/auxiliary role for platelet P-selectin) and involves SLC44A2 on neutrophils (*16*). Consistent with this, mice deficient in SLC44A2 are protected against experimental venous thrombosis (*17*–*19*). Moreover, *SLC44A2* is a susceptibility locus for venous thromboembolism (VTE) due to a prevalent single-nucleotide polymorphism (SNP; rs2288904; minor allele frequency of 0.22) encoding a R154Q substitution that disrupts α_IIb_β_3_ binding and protects against VTE (*16*, *20*). This SNP protects against VTE with individuals homozygous for *SLC44A2*(R154Q) having 30 to 50% reduced incidence of VTE (*20*, *21*).

Together, these data are consistent with a model in which VWF-dependent capture of platelets to the venous endothelium specifically augments neutrophil recruitment and subsequent activation. However, there remain conceptual issues with this model that require resolution. First, in the absence of endothelial damage/vessel injury, how does VWF recruit platelets to the venous valve pocket? Second, given that VWF function is augmented by elevated shear (*22*–*24*), how effectively can endothelial VWF recruit platelets at low/aberrant venous shear rates associated with valve pockets? Third, if VWF strings secreted by the endothelium recruit platelets, then why are these not controlled/prevented by ADAMTS13-dependent proteolysis (*25*, *26*)? Fourth, how do VWF-bound platelets specifically recruit neutrophils (as opposed to other leukocytes) in the absence of classical platelet agonists associated with blood vessel damage? Therefore, to explore the molecular and cellular mechanisms responsible for the initiation of venous thrombosis, we modeled and interrogated these processes using endothelialized fluidic systems.

## RESULTS

### Shear-dependent platelet/neutrophil recruitment

**Fig. 1.**
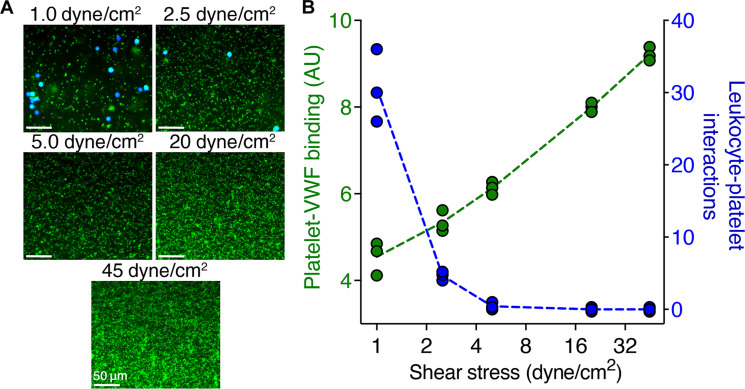
Shear dependence on VWF of platelet and platelet-dependent neutrophil recruitment. (**A**) Representative images of platelets binding to immobilized VWF following 3-min perfusion of PFB at 1 to 45 dyne/cm^2^. Platelets and leukocytes both stained with DiOC6 (green) and leukocytes (pseudo-colored blue) were mapped using the Slidebook software to distinguish cells. Scale bars, 50 μm. (**B**) Quantification of platelet binding (green, left axis) and mean number of bound leukocytes after 3 min across 10 fields of view (blue right axis) as a function of shear. Data presented are mean values from 10 distinct fields of view through each channel (*n* = 3). AU, arbitrary unit.

Platelet binding to VWF is a shear-dependent process. Shear stress is not only required to unravel VWF to enable platelet binding, but the glycoprotein (GP)Ibα–VWF A1 domain interaction that ensues is also augmented by elevated shear forces (*22*). This has led to the contention that VWF function is more important at arterial than venous shear stress, although numerous studies support a role for VWF in the pathogenesis of venous thrombosis (*27*–*31*). To explore this, we measured platelet binding to VWF (immobilized on linear fluidic channels) at different shear stresses (venous, 1 to 6 dyne/cm^2^; arterial, 10 to 45 dyne/cm^2^) (Fig. 1). As shear stress increased, there was an increase in platelet recruitment at 3 min after perfusion of platelets. This may be due to the increased number of platelets perfused at 3 min as shear rate increases. The differences between these shear stresses were largely lost when we perfused the same volume of plasma-free blood (PFB) (i.e., number of platelets) over the VWF surface at different shear stresses (fig. S1). We also noted that at lower “venous shear” stress (1 to 5 dyne/cm^2^) that leukocyte interactions with platelets occurred. These diminished as a function of increasing shear stress (Fig. 1) and were not detected at “arterial” shear stresses, suggesting that these platelet-leukocyte interactions may be more relevant to the venous system as they are unable to withstand the forces associated with arterial flow.

### Shear-dependent VWF-platelet string formation

**Fig. 2.**
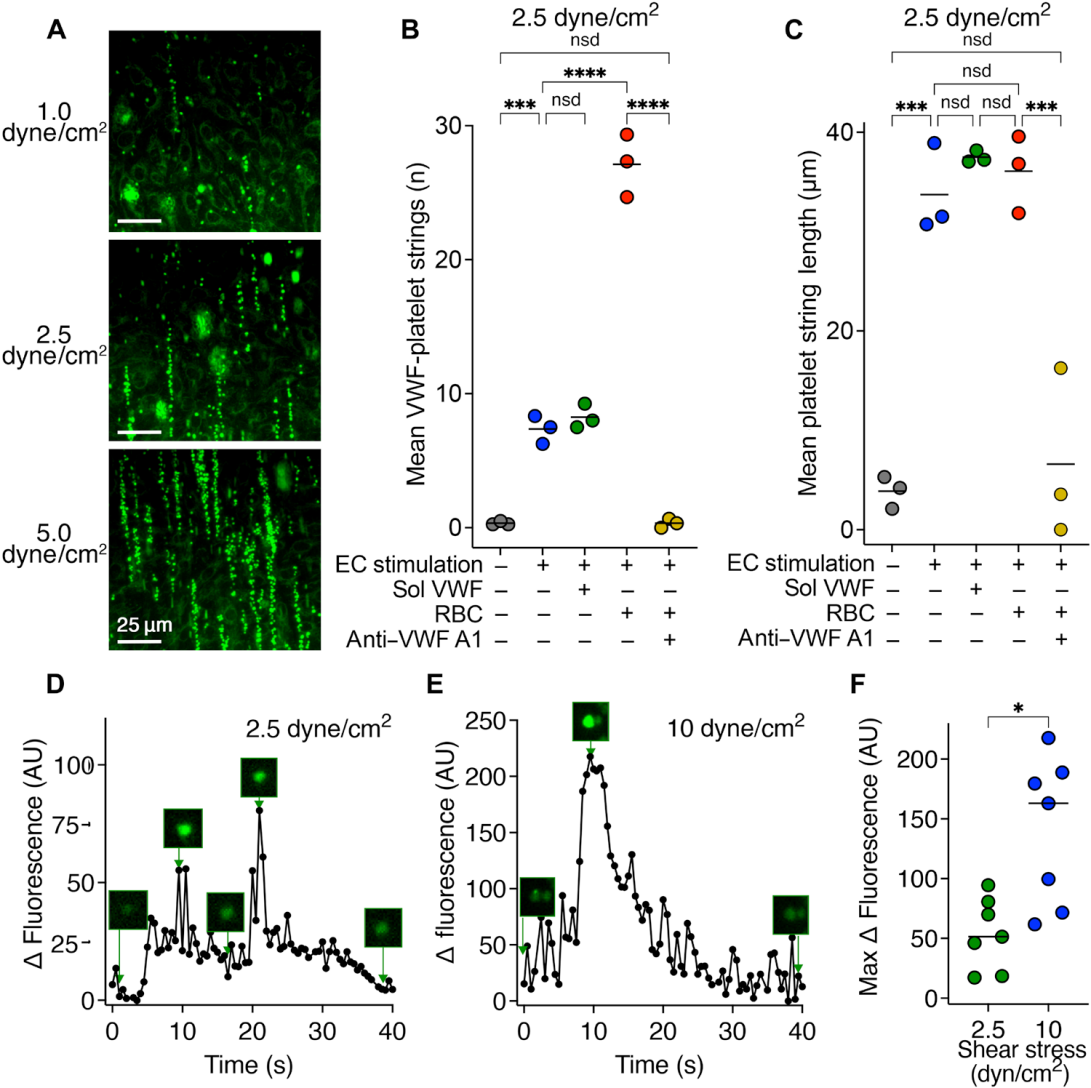
Shear-dependent platelet binding and signaling on VWF strings. (**A**) PFB stained with DiOC6 to label platelets (green) was perfused over HUVECs stimulated with 100 μM histamine/10 μM adrenaline. Images shown are platelet-VWF strings at 1.0, 2.5, or 5.0 dyne/cm^2^ at 2 min after initiation of perfusion. Scale bars, 25 μm. (**B**) Number of VWF-platelet strings formed over ECs after 2 min of washed platelet perfusion ± histamine/adrena (top) (1.0 dyne/cm^2^) also presented in fig. S2 as a comparator. Line stimulation, soluble (sol) VWF, RBCs, and/or an anti–VWF A1 domain antibody that blocks platelet binding to VWF. Each data point represents the mean of 10 fields of view with the mean of *n* = 3 shown. (**C**) Length of VWF-platelet strings across 10 fields of view in each channel condition as in (B). Note string length in the presence of anti–VWF A1 domain antibody represents mean of very occasional/rare strings. Data presented are the mean of *n* = 3. (**D** and **E**) Representative trace and images of intraplatelet Ca^2+^ release within platelets bound to VWF strings at 2.5 or 10 dyne/cm^2^. Platelets containing Ca^2+^-sensitive dye, Fluo-4AM, were perfused over stimulated HUVECs at 2.5 or 10 dyne/cm^2^. Change in fluorescence of individual VWF-bound platelets was monitored over 40 s. Individual platelets were isolated, and the change in fluorescence over 40 s period was measured. See movie S1 for example at 10 dyne/cm^2^. (**F**) Maximum change in Fluo-4AM fluorescence from baseline of individual VWF-bound platelets undergoing Ca^2+^-signaling events at 2.5 or 10 dyne/cm^2^. All data points are presented (*n* = 7) with the median shown. Data in (B) and (C) were compared by one-way analysis of variance (ANOVA) with Tukey’s multiple comparison test; data in (F) were compared by an unpaired two-tailed Mann-Whitney test; *P* values < 0.05 were considered significant. nsd, not significantly different. *P < 0.05, ***P < 0.001, and ****P < 0.0001.

Immobilization of VWF to fluidic channel surfaces causes the exposure of the VWF A1 domain that enables platelet binding. Therefore, to explore the influence of shear upon both VWF unraveling and subsequent platelet recruitment, we examined the formation of VWF-platelet strings over the surface of confluent human umbilical vein endothelial cells (HUVECs) cultured in fluidic channels. Perfusing PFB at venous-level shear stress (1 to 5 dyne/cm^2^) over HUVECs that were prestimulated for 5 min with 100 μM histamine/10 μM adrenaline to induce acute VWF release revealed a shear-dependent increase in VWF-platelet string formation (Fig. 2A) that was still evident when we looked at strings formed at different shear stresses after the same volume of PFB had been perfused (fig. S2). Despite this, VWF-platelet string formation still occurred even at the lowest shear stress tested. To explore this further, we examined the influence of HUVEC stimulation, soluble VWF, red blood cells (RBCs), and VWF blockade on both the number and length of VWF-platelet strings. In the absence of prestimulation of HUVECs with 100 μM histamine/10 μM adrenaline, only a small number of short strings were evident (Fig. 2, B and C). As expected, HUVEC stimulation significantly increased the number and length of VWF-platelet strings at 2.5 dyne/cm^2^. These parameters were not influenced by the presence of soluble VWF, suggesting that string formation does not require/is not augmented by soluble VWF at venous shear (Fig. 2, B and C). The presence of RBCs appreciably increased the number of VWF-platelet strings, likely associated with their marginating effect upon platelets as previously reported (*32*), but this did not influence string length. As expected, blockade of the VWF A1 domain to prevent VWF-platelet binding markedly reduced platelet string formation (Fig. 2, B and C).

### VWF-dependent mechanosensitive signaling

When platelets bind to immobilized VWF at arterial shear stress (~45 dyne/cm^2^), they undergo repeated release of intraplatelet Ca^2+^ (*16*) due to mechanosensitive signal transduction through GPIbα (*33*). Given that signal transduction is shear dependent, the magnitude of signaling may be influenced by shear stress. Preloading platelets in PFB with Ca^2+^-sensitive fluorophore, Fluo-4AM, enabled us to visualize changes in intraplatelet Ca^2+^ release in VWF string-bound platelets in real time at different shear stresses (2.5 and 10 dyne/cm^2^) (Fig. 2, D to F, and movie S1). These data revealed that mechanosensitive signaling occurred at venous shear stress. The intensity of Ca^2+^-dependent fluorescence was significantly higher at increased shear stress, consistent with the mechanosensitive nature of signal transduction (*33*).

### Neutrophil recruitment/activation on VWF-platelet strings

**Fig. 3.**
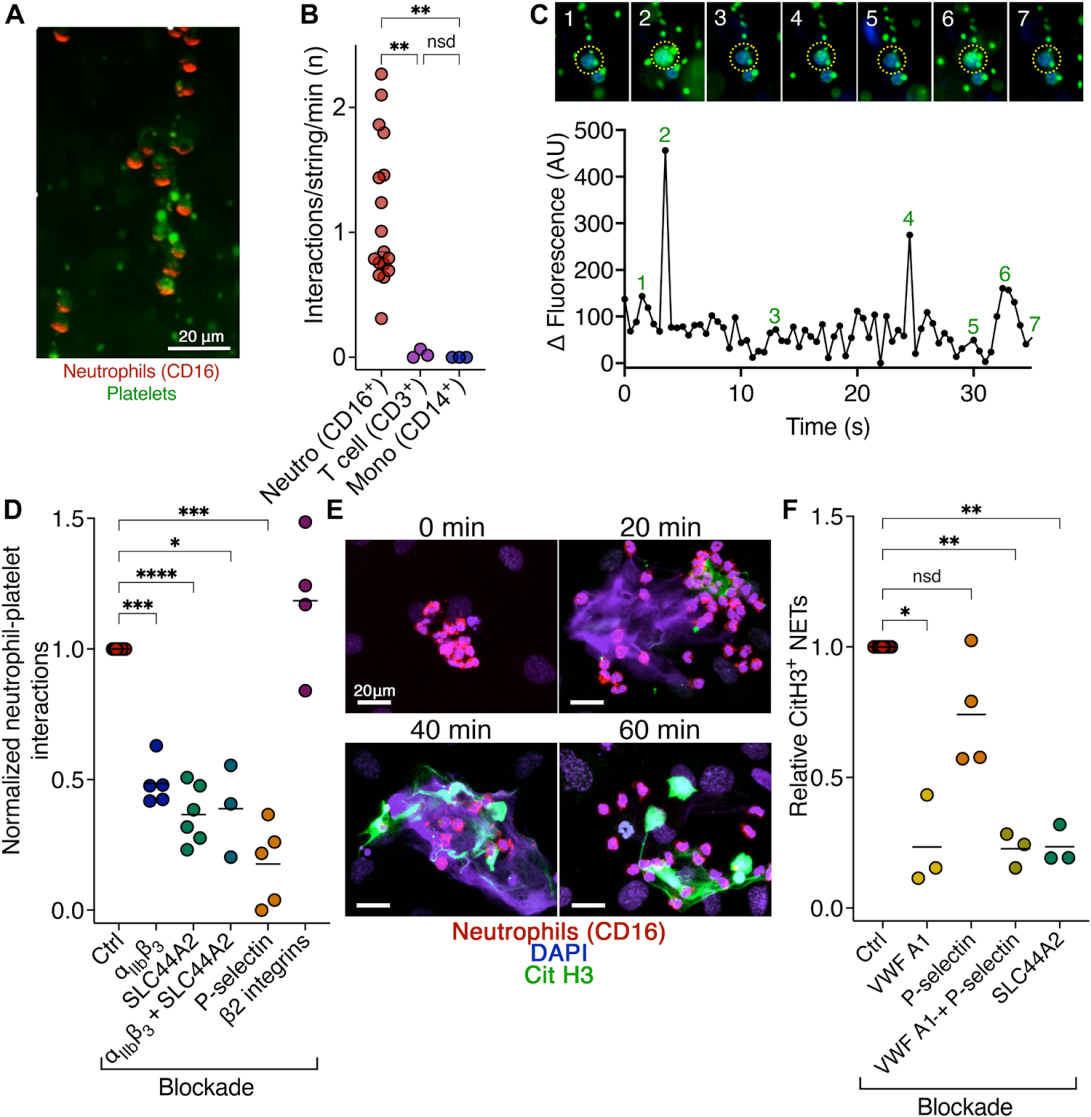
Neutrophil binding, signaling and NET formation on VWF-bound platelet strings. (**A**) Washed platelets (green) were perfused over stimulated HUVEC at 2.5 dyne/cm^2^ for 2 min to form VWF-platelet strings. Isolated leukocytes containing neutrophils (anti-CD16, red) were perfused at 0.5 dyne/cm^2^ for 6 min. Scale bar, 20 μm. See movie S2. (**B**) Leukocyte interactions with VWF-platelet strings performed as in (A) except PBMCs (containing monocytes and T cells) or granulocytes (containing neutrophils) were isolated and labeled with anti-CD16 (neutrophils), anti-CD14 (monocytes), or anti-CD3 (T cells). Cells were perfused over VWF-platelet strings for 15 min. *n* = 3 to 17. (**C**) Neutrophils preloaded with Fluo-4AM at 0.5 dyne/cm^2^ were perfused over VWF-platelet strings. Changes in fluorescence of individual platelet bound neutrophils were monitored over 35 s. (**D**) Neutrophils and VWF-platelet string interactions performed as in (A) in the presence or absence of GR144053, anti-SLC44A2 antibody, anti–P-selectin, or anti-β_2_ antibody. Normalized interactions/VWF-platelet string/min is presented relative to the paired control (no block). Data presented are means ± SD (*n* = 3 to 8). (**E**) Neutrophils and NETs bound to VWF-platelet strings and ECs, following incubation for 0 to 60 mins post-perfusion of blood. Neutrophils (red) were perfused for 10 min over VWF-platelet strings. Unbound neutrophils were washed, and channels were incubated for 0 to 60 min before fixation and staining for CitH3 (green) and DNA (DAPI, blue). (**F**) Number of CitH3-positive NETs at 60 min in the presence or absence of anti–VWF A1 domain antibody, anti–P-selectin (or both), or anti-SLC44A2 antibody. CitH3-positive NETs were normalized to the number of NETs within the corresponding paired control (*n* = 3 to 8). Data in (B) were compared by Kruskal-Wallis multiple comparison test; (D) and (F) were compared by one-way ANOVA with Dunnett’s multiple comparison test. *P* values < 0.05 were considered significant. *P < 0.05, **P < 0.01, ***P < 0.001, and ****P < 0.0001.

Neutrophils bind specifically to platelets bound to VWF adsorbed onto fluidic channels (*16*). To assess whether platelets bound to VWF strings under venous shear were also capable of specific recruitment of neutrophils, we stimulated HUVECs in microchannels with 100 μM histamine/10 μM adrenaline and perfused washed platelets at 2.5 dyne/cm^2^ for 2 min to create strings. Thereafter, we perfused (at 0.5 dyne/cm^2^) either isolated granulocytes labeled with anti-CD16 (neutrophils) or isolated peripheral blood mononuclear cells (PBMCs) labeled with either anti-CD3 (T cells) or anti-CD14 (monocytes) (Fig. 3, A and B). Using this approach, neutrophils were the primary leukocyte interacting with VWF-bound platelets (movie S2), with a small number of occasional T cell interactions also observed. Monocyte interactions with VWF-bound platelets were not detected.

In similar assays performed using isolated granulocytes preloaded with Fluo-4AM, we measured transient release of intraneutrophil Ca^2+^ stores following binding to VWF-platelet strings, indicative of a signal being transduced into the neutrophil after binding to VWF-primed platelets under low venous shear stress (Fig. 3C).

Using blocking agents against activated α_IIb_β_3_ (GR144053) or SLC44A2 (anti-SLC44A2), we could diminish neutrophil interactions with VWF-platelet strings by 50 to 60% (Fig. 3D) during the first 6 min of perfusion, consistent with our previous findings that activated α_IIb_β_3_ can recruit neutrophils via an interaction that is dependent on neutrophil SLC44A2 (*16*). In agreement with their codependency, we detected no synergistic effect of simultaneously blocking both α_IIb_β_3_ and SLC44A2 (Fig. 3D). Blocking β_2_ integrins (i.e., lymphocyte function-associated antigen 1/α_L_β_2_ and macrophage-1 antigen/α_M_β_2_) with an anti-CD18 antibody had no effect on neutrophil binding to VWF-platelet strings. Conversely, blocking P-selectin reduced neutrophil binding by ~75% during the first 6 min of perfusion. Previously, we reported that blocking P-selectin on platelets bound to immobilized VWF on a channel surface had minimal influence on neutrophil binding, although we detected an increase in neutrophil rolling speed, suggesting an auxiliary role of platelet P-selectin (*16*). However, in the present assay that included an endothelial cell surface, we measured an increased contribution suggesting that P-selectin on the endothelial cell surface trafficked during VWF release may contribute to the efficacy of neutrophil recruitment to the VWF-platelet strings.

To further explore the influence of VWF-platelet–dependent recruitment of neutrophils, we examined neutrophils at different time points (0 to 60 min) after recruitment for evidence of NET formation (Fig. 3E). Extracellular DNA was observed as early as 20 min and positive staining for citrullinated histone H3 (CitH3) from 40 min, suggesting that neutrophil capture to VWF-bound platelet strings under low venous shear stress transduces a signal into the neutrophil that can drive NET formation. To assess the contribution of different receptors to NET formation, we blocked platelet binding to VWF, which resulted in a marked reduction in NET formation at 60 min by ~75% (Fig. 3F). We detected a similar effect when SLC44A2 was blocked, consistent with a major role of both platelets and neutrophil SLC44A2 in NET formation. The effect of blocking P-selectin on the number of neutrophils that formed NETs after 60 min was not statistically different from the control, suggesting that although P-selectin may play a role in process of early neutrophil recruitment itself, it plays a less important role in the transduction of a signal that drives subsequent NET formation (Fig. 3F).

### Aberrant flow and ADAMTS13-resistant VWF tangles

**Fig. 4.**
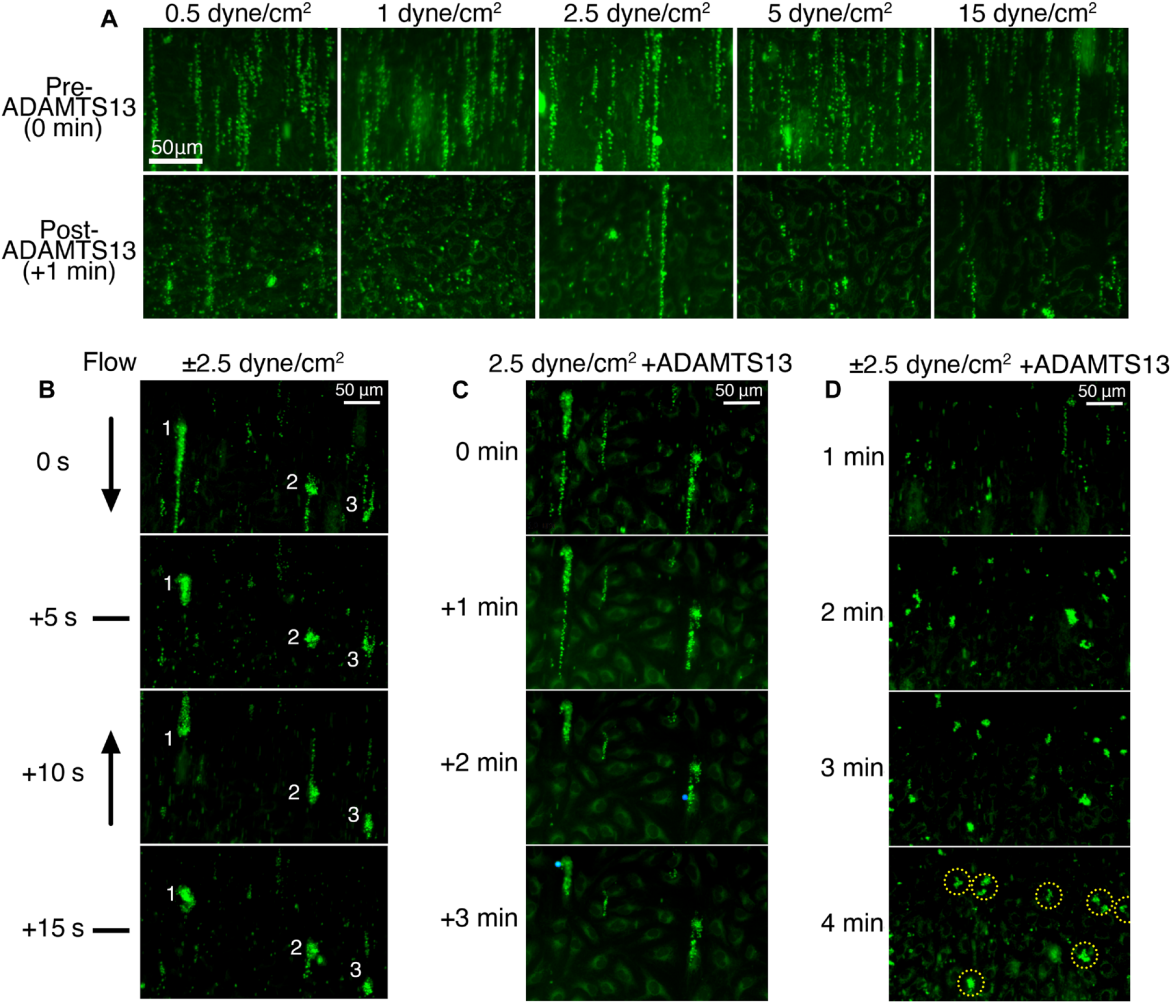
VWF-platelet string tangling promotes resistance to ADAMTS13-mediated VWF proteolysis. (**A**) Images of ADAMTS13-mediated VWF-platelet string proteolysis as a function of shear stress. VWF-platelet strings were generated through perfusion of DiOC6-labeled PFB at 5 dyne/cm^2^ in the absence of ADAMTS13 over HUVECs stimulated with 100 μM histamine/10 μM adrenaline. After 2 min of blood perfusion (top panels), 50 nM ADAMTS13 in HT-BSA buffer was perfused at 0.5 to 15 dyne/cm^2^, and proteolysis/VWF-platelet string removal was observed after 1 min (bottom). Scale bar, 50 μm. Images representative of *n* = 3. (**B**) Representative images (*n* = 3) of the formation of VWF-platelet tangles on the EC surface. Following the stimulation of HUVECs with 100 μM histamine/10 μM adrenaline, PFB was perfused at 2.5 dyne/cm^2^ for 1 min under unidirectional flow to form strings. Thereafter, VWF-platelet string tangles were created by perfusing PFB forwards and backwards at ±2.5 dyne/cm^2^ (±400 μl/min, wave period = 10 s) for 5 min. Individual VWF-platelet strings (*1*–*3*) are labeled to highlight changes in string structure as a function of change in flow directionality. Panels show the changes in strings/tangles through a single period of 15 s during the flow reversal cycle. see movie S3. (**C**) Following generation of VWF-platelet string tangles in (B) the ability of ADAMTS13 to proteolytically remove VWF-platelet tangles was assessed. A total of 50 nM ADAMTS13 was perfused over the channel at 2.5 dyne/cm^2^ for 3 min. (**D**) Representative images of the formation of VWF-platelet tangles over HUVECs stimulated with 100 μM histamine/10 μM adrenaline in the presence of 5 nM ADAMTS13. PFB containing 5 nM ADAMTS13 was perfused at ±2.5 dyne/cm^2^ as in (C), and platelet-VWF binding was monitored. See movie S3. At 4 min, ADAMTS13 resistant VWF-platelet tangles are circled in yellow. Platelets stained with DiOC6 (green). See movie S4. Images are representative of *n* = 3.

All the VWF-platelet string assays performed so far were performed in the absence of plasma meaning that ADAMTS13, the plasma metalloprotease that normally proteolyzes VWF to release VWF strings from the endothelial surface, was not present (*34*). To explore the shear dependence of ADAMTS13 function, we first created VWF-platelet strings by perfusing PFB over histamine/adrenaline-stimulated HUVECs at 5 dyne/cm^2^. Thereafter, we perfused 50 nM ADAMTS13 over the strings at 0.5 to 15 dyne/cm^2^ and monitored platelet string removal (Fig. 4A). At all shear stresses, ADAMTS13 efficiently proteolytically cleared the VWF-platelet strings after 1 min, revealing that even at low venous shear stress the forces exerted on VWF by flow are adequate to induce VWF A2 domain unfolding required for ADAMTS13 proteolysis. In some cases, strings persisted. These generally corresponded to more complex VWF-platelet strings/cables. That some of these string/cables persisted for a little longer suggested that more complex VWF-platelet strings involving strings coalescing or tangling might be less efficiently proteolyzed by ADAMTS13.

These assays were all performed using unidirectional flow. Whereas this flow is relevant to most of the vasculature, it is less representative of the flow patterns that may occur in the venous valve pocket where DVT develops. Because of the role of venous valves in preventing blood reflux, blood flow in these locations likely involves multidirectional flow. As a first attempt to mimic this, we generated VWF-platelet strings at 2.5 dyne/cm^2^ as before and then exposed the strings to oscillatory (bidirectional but uniaxial) flow for 5 min that caused the strings to tangle by a repeated cycle of forward and then reverse flow in a 10 s period reaching 2.5 dyne/cm^2^ in each direction (Fig. 4B and movie S3). This flow pattern caused the VWF strings to tangle into more complex knotted structures. When we subsequently perfused 50 nM ADAMTS13 through these channels unidirectionally at 2.5 dyne/cm^2^, these complex knotted VWF-platelet tangles were appreciably more resistant to ADAMTS13-mediated proteolysis (Fig. 4C). This finding corroborated the suggestion that VWF-platelet string tangling caused by multidirectional flow, similar to what may occur in venous valve pockets, has the potential to enable persistence of VWF-bound platelets that can resist ADAMTS13.

We next repeated this assay using PFB that contained physiological ADAMTS13 concentrations (5 nM) from the start. Under these conditions, the size of the VWF-platelet tangles that we observed were smaller but despite the presence of ADAMTS13 from the start, we detected multiple tightly knotted VWF-platelet tangles after 4 min that persisted on the surface of the endothelial cells (Fig. 4D and movie S4).

### VWF string/tangle formation

**Fig. 5.**
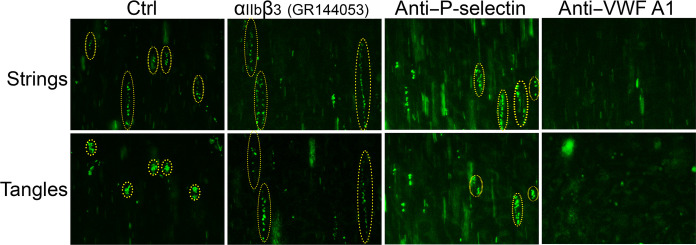
Influence of α_IIb_β_3_, P-selectin, and VWF A1 domain on VWF-platelet string and tangle formation. Representative images of VWF-platelet strings through perfusion of DiOC6-labeled PFB under unidirectional flow at 5 dyne/cm^2^ for 2 min over confluent HUVECs stimulated with 100 μM histamine/10 μM adrenaline. PFB was perfused in the absence (Ctrl) or presence of GR144053 (α_IIb_β_3_ blocker), anti–P-selectin or anti–VWF A1 domain (top panels). Thereafter, VWF-platelet string tangles were created in the same field of view by perfusing PFB forward and backward at ±5 dyne/cm^2^ (±1.2 ml/min, wave period = 10 s) for 2.5 min (bottom panels). Images are representative of *n* = 3.

To further explore modifiers of VWF string and tangle formation, we repeated assays under similar conditions in the presence of different blocking agents. As before VWF-platelet strings formed over the surface of stimulated HUVECs under unidirectional flow, which then knotted into tangles during multidirectional flow (Fig. 5). In the presence of GR144053, which blocks activated α_IIb_β_3_, string formation appeared normal. However, the efficiency of tangle formation appeared reduced, with strings more frequently persisting in a linear form and knots forming less efficiently. This may suggest that α_IIb_β_3_ activation (which enables further VWF binding) assists in the formation of tangles. Blocking P-selectin appeared to have little effect on either string or tangle formation. As expected, blocking the VWF A1 domain to inhibit GPIbα binding prevented VWF-platelet string formation completely. To ascertain whether string and tangle formation was influenced by endothelial cells source, we performed the same experiments using human pulmonary microvascular endothelial cells (HMVECs) and human aortic endothelial cells (HAECs). VWF-platelet strings formed over HMVEC, similar to HUVEC, and tangle formation was similarly diminished using GR144053 (fig. S3). Using HAEC, we detected an appreciable increase in VWF-platelet string formation (fig. S4), potentially due to increase VWF expression/secretion from the HAEC. These strings frequently coalesced with adjacent strings with multiple attachment points during multidirectional flow causing the tangles to remain more elongated rather than knotting into foci. Similar results were observed when P-selectin was blocked. Blocking α_IIb_β_3_ with GR144053 did not inhibit tangle formation or string coalescence as effectively as over HUVEC (Fig. 5) or HMVEC (fig. S3) when string density was lower. This suggests that α_IIb_β_3_ activation following GPIbα binding to VWF under flow can contribute to tangle formation, but that this process is more highly dependent upon VWF and GPIbα.

### Fluidic venous valve channel

**Fig. 6.**
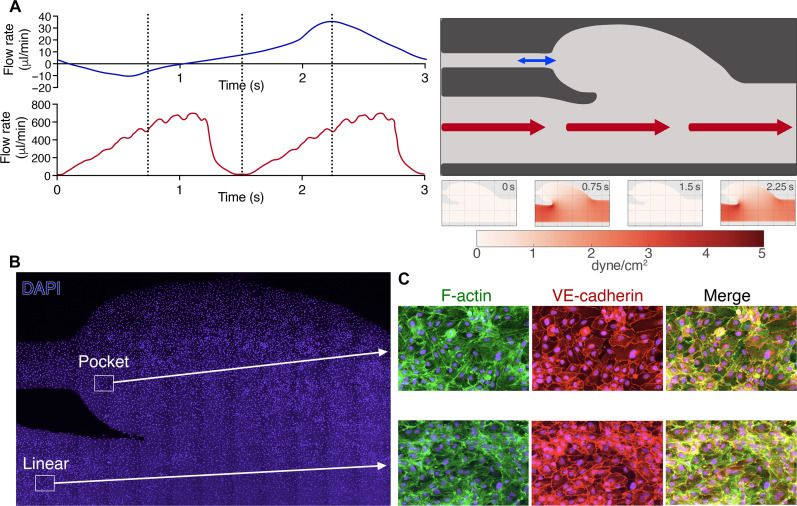
Development of a fluidic venous valve model. (**A**) Representative diagram of the venous valve fluidic channel (top right) developed to mimic aberrant flow patterns associated with VTE-prone valves (for channel dimensions, see fig. S3). Flow through the main part of the channel was pulsatile (red arrows) with a 1.5-s period leading to shear stress up to 2.5 dyne/cm^2^ in the linear part of the channel. Flow rate represented by the red line graph. To create low aberrant flow within the valve pocket and to prevent passive accumulation of blood cells, a low oscillatory flow pattern with a 3-s period (note *y*-axis scale, blue arrow) was introduced through the inlet in the valve pocket—flow rate through the valve inlet channel represented by the blue line graph. Computational fluid dynamics (CFD) shown below channel representing changes in the wall shear stress within the valve channels at 0, 0.75, 1.5, and 2.25 s (denoted by vertical dashed lines) conducted using OpenFOAM during perfusion of a Newtonian fluid. (**B**) HUVECs were seeded and cultured in venous valve channels with a shear stress of 5 dyne/cm^2^ in the linear part of the channel for 48 hours before experimentation to ensure formation of a confluent monolayer of cells. DAPI staining depicts cell nuclei. (**C**) Staining for F-actin (green) and VE-cadherin (red) in both the linear and pocket sections of the channel was used to ensure confluency and formation of a contiguous cell monolayer in both the linear and pocket regions of the channel.

**Fig. 7.**
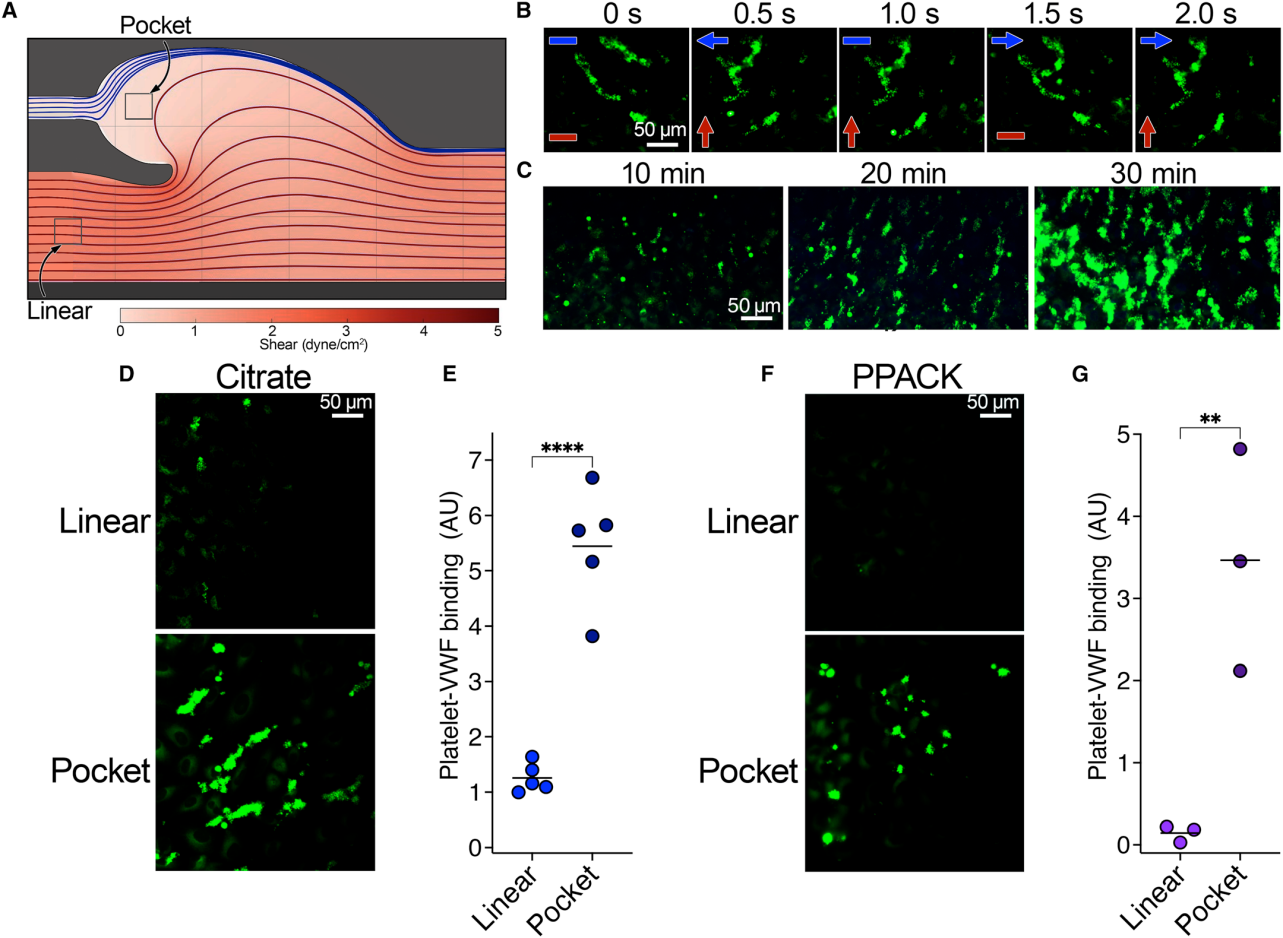
Aberrant flow patterns in venous valve pocket enable VWF-dependent platelet recruitment to endothelial surfaces. (**A**) CFD simulation of streamlines to visualize the flow patterns in the venous valve channel (representation at 2.5 s in the 3-s period shown in Fig. 6A). The boxed pocket and linear parts of the channel correspond to the regions imaged/analyzed in (B) to (G). For changes in flow direction in the pocket during experiments see fig. S4. (**B**) VWF-platelet tangles in the valve pocket. HUVECs cultured for 2 days under flow were stimulated with 100 μM histamine/10 μM adrenaline for 5 min. Citrated whole blood labeled with DiOC6 to label platelets (green) was perfused through channels. Images of VWF-platelet tangle formation after 15 min over a 3-s cycle, highlighting how multidirectional flow affects tangling of structures in the valve pocket. Red arrows highlight overall direction of blood perfusion from the main channel, and blue arrows indicate fluid direction from the valve inlet (see movie S5). (**C**) Images of VWF-platelet tangles in the pocket of the valve channels at 10, 20, and 30 min from initiation of perfusion of whole blood. (**D**) Images of VWF-platelet tangle formation in the linear and pocket regions of venous valves channels. Platelets (green) 15 min after initiation of citrated whole blood perfusion. (**E**) Mean number of VWF-platelet strings/tangles formed on ECs in the linear/pocket regions of venous valve channels. Platelet capture was quantified using the mean sum fluorescence across 10 fields of view in both linear and pocket regions of the channel and the means shown (*n* = 5). (**F**) As in (D) except using PPACK-anticoagulated blood. (**G**) As in (E) except using PPACK-anticoagulated blood (*n* = 3). Data in (E) and (G) were analyzed using an unpaired two-tailed *t* test. *P* values < 0.05 were considered significant. **P < 0.01 and ****P < 0.0001.

To endeavor to better mimic the aberrant flow patterns that may exist in and around venous valves of individuals with stasis-related risk factors for DVT, we developed a fluidic channel that incorporated a valve pocket (Fig. 6A and fig. S5). Flow was controlled through the main channel with one fluidic pump enabling pulsatile unidirectional flow to create mean flow at levels that approximate the shear stresses that are associated with venous flow (5 dyne/cm^2^) in the linear part of the channel—representing the effect of normal blood flow. In addition, we inserted a channel to the base of the valve pocket to gently introduce a multidirectional flow pattern at much lower shear stress when connected to a second fluidic pump. We hypothesized may mimic the aberrant flow that may exist in the deep veins of individuals with stasis-related risk factors for VTE (fig. S6) and prevent passive collection of cells in the pocket due to the lack of flow. HUVECs were cultured in these channels under unidirectional pulsatile venous flow (only through the main channel) for 2 days to enable a confluent monolayer of endothelial cells to form throughout the channel (Fig. 6, B and C). To assess platelet binding to VWF secreted by the endothelial cells, we stimulated the endothelial cells in the channel with 100 μM histamine/10 μM adrenaline for 5 min to induce VWF secretion before perfusion of citrated whole blood and monitored platelet binding to VWF in both the linear and valve pocket regions of the channel (Fig. 7A). Using this approach, we specifically detected complex knotted VWF-platelet tangles within the pocket region of the channels. A complex VWF-platelet tangle is shown in Fig. 7B over a 2-s cycle showing the movement of the tangle during changes in flow from the main channel flow (red arrow) and the valve inlet channel (blue arrow) (movie S5). Accumulation of VWF-platelet tangles continued over an extended period (up to 30 min) (Fig. 7C), which included recruitment of leukocytes [also stained with 3,3′-dihexyloxacarbocyanine iodide (DiOC6)] to the VWF-platelet tangles. To gauge the relevance/specificity of VWF-platelet recruitment to the valve pocket, we also analyzed platelet recruitment in the linear part of the channel (Fig. 7, D and E). Because of the use of whole blood, ADAMTS13 was present at physiological concentrations. Therefore, under these conditions, the linear parts of the channel were essentially devoid of platelets, likely due to the efficient removal of strings by ADAMTS13. We did detect occasional small platelet strings in the linear part of the channel. The use of citrate as an anticoagulant depletes free Ca^2+^ in plasma. ADAMTS13 is a Ca^2+^-dependent metalloprotease (*35*). Although the removal of Ca^2+^ does not completely inactivate the enzyme, it does diminish its enzymatic function (*35*). We therefore repeated this assay using whole blood anticoagulated with d-phenylalanyl-l-prolyl-l-arginine chloromethyl ketone (PPACK). Under these conditions, the linear portion of the channel was completely devoid of VWF-bound platelets, whereas the valve pockets continued to accumulate VWF-platelet tangles, albeit with modestly reduced size when compared to citrated blood (Fig. 7, F and G). These data strongly support the contention that aberrant, multidirectional flow patterns within the valve pocket can result in ADAMTS13-resistant VWF-platelet tangles that enable these knotted cables to persist.

### VWF-platelet tangle and neutrophils recruitment in venous valve channel

**Fig. 8.**
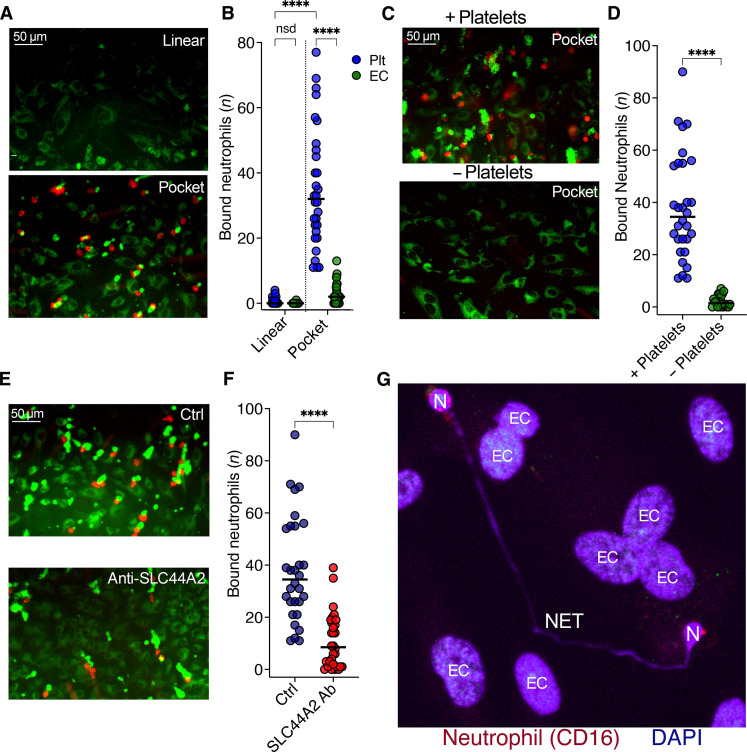
Platelet-dependent neutrophil recruitment to the venous valve pocket. (**A**) Neutrophil capture on VWF-bound platelets/HUVECs within the linear and pocket regions of venous valve channels. Citrated whole blood (platelets, green) was perfused for 15 min after stimulation followed by perfusion of neutrophils (red) for 5 min. (**B**) Channels were washed and neutrophils bound to either VWF-platelet tangles (green) or HUVEC (i.e., not associated with platelets, blue) in the linear, and pocket regions were counted. Neutrophils in 10 fields of view are presented (*n* = 3), and the median is shown. (**C**) Neutrophils bound to VWF-platelet tangles and HUVECs in the pocket of venous valve channels, performed in the presence/absence of platelets. (**D**) Number of bound neutrophils in each field of view, in the presence or absence of platelets, was counted across 10 fields of view (*n* = 3), and the median is shown. (**E**) Neutrophils bound to VWF-platelet tangles in the pocket of venous valve channels, in the absence (Ctrl) or presence of anti-SLC44A2 blocking antibody. (**F**) Number of bound neutrophils in each field of view, in the absence or presence of SLC44A2 blockade, was counted across 10 fields of view, and the median is shown (*n* = 3). Ab, antibody. (**G**) Image of neutrophils releasing extracellular DNA within the pocket of venous valve channels. Experiments were performed as in (A). Neutrophils (red) were then perfused through the channel for 5 min, unbound cells were washed out, and DNA was visualized by staining with DAPI (blue) after 40 min. Extracellular DNA (NET) released from neutrophils (N) were observed within the pocket region and the nuclei of the underlying HUVECs (EC). Data in (B) were compared by nonparametric Kruskal-Wallis multiple comparison test; (D) and (F) were compared with an unpaired two-tailed Mann-Whitney test. *P* values < 0.05 were considered significant. ns, not significant. ****P < 0.0001.

To assess the specific recruitment of neutrophils to VWF-platelet tangles within the venous valve channel, we perfused whole blood through 100 μM histamine/10 μM adrenaline stimulated HUVECs for 15 min to accumulate VWF-platelet tangles. Specific labeling of neutrophils in whole blood was not possible due to the fluorescence quenching of the signal by the RBCs. Therefore, after 15 min, channels were washed out at venous shear before perfusion of isolated granulocytes labeled with anti-CD16 to identify neutrophils for 5 min. Thereafter, the number and distribution of neutrophils were quantified through the channel (Fig. 8, A and B). Neutrophil binding in the linear part of the channel was minimal with those detected being primarily associated with VWF-bound platelets. Within the valve pocket appreciably higher numbers of neutrophils were detected, the majority of which were associated with VWF-platelet tangles in this region. Some neutrophils were also observed without VWF-platelet tangles, suggesting that they may be directly interacting with the endothelial cells. Consistent with this, when we repeated these experiments comparing whole blood with reconstituted whole blood without platelets, we detected significantly fewer neutrophils in the valve pockets when platelets were absent, consistent with the contention that neutrophil recruitment in this system is highly dependent on VWF-bound platelets (Fig. 8, C and D) and argues against direct binding of neutrophils with VWF.

To explore the dependency of neutrophil recruitment on SLC44A2 binding to the VWF-bound platelets within the valve pocket, we performed experiments in the presence of anti-SLC44A2 antibodies, which resulted in a significant (~75%) reduction in median neutrophil binding to VWF-bound platelets (Fig. 8, E and F), consistent with the data in linear channels (Fig. 3D). Similar to the linear channels, evidence of NET formation by neutrophils bound within the valve pocket became evident after ~40 min (Fig. 8G).

## DISCUSSION

In this study, we endeavored to provide mechanistic insight into the initiation of venous thrombosis, with an emphasis on understanding the role of VWF, and how this might explain the proclivity for thrombosis to develop in venous valve pockets. The role of VWF in venous thrombosis is perhaps less obvious given the lack of vessel damage and the low shear environment. Despite this, there is a positive association of plasma VWF with the incidence of venous thrombosis (*27*–*30*), which is even stronger when considering unprovoked DVT (*31*). Part of this association can be attributed to the association of VWF levels with those of plasma factor VIII, which is a positive modifier of venous thrombosis risk (*36*, *37*). Despite this, the platelet-binding function of VWF appears to be of primary importance in venous thrombosis initiation. Brill and colleagues (*15*) elegantly demonstrated that blocking VWF in mice protects against venous thrombus formation in the stenosis model and that *Vwf*^−/−^ mice infused with factor VIII were still protected against thrombus formation. A further and important observation was the finding that both platelet and leukocyte recruitment to the venous vessel wall was almost completely abolished by VWF deficiency (*15*) underscoring the primary importance of VWF-dependent platelet recruitment and the role of these platelets in subsequent leukocyte recruitment (*12*, *15*).

A pathogenic role of VWF in venous thrombosis presents certain conceptual issues. First, how effectively does VWF recruit platelets at low venous shear? Using both immobilized VWF and VWF strings secreted from endothelial cells, we show that platelets bind effectively at low venous shear (Figs. 1 and 2A and figs. S1 and S2). For VWF to contribute to venous thrombosis in the absence of overt endothelial damage, it seems highly likely that platelet capture is mediated by the formation of VWF strings/cables that can ensue following VWF release. This puts a greater emphasis on the cellular, rather than plasma, pool of VWF. A second major question with a role of endothelial secreted VWF strings is that ADAMTS13 normally efficiently clears these from the endothelial surface even at low shear stress (Fig. 4A). We found that VWF strings/cables on different endothelial cell types that were exposed to multidirectional flow tangled into VWF-platelet knots (Fig. 4, C and D and 5, and figs. S3 and S4). The ability of VWF-platelet strings to form tangles involved both GPIbα and activated α_IIb_β_3_, which can both contribute to further VWF binding. These aberrant flow patterns are more likely to occur in the venous valve pocket of individuals with stasis-related risk factors, whereas elsewhere in the vasculature, flow will be primarily unidirectional meaning that these VWF-platelet knots are unlikely to develop. Of particular importance was the finding that VWF-platelet tangles exhibited appreciable resistance to ADAMTS13-dependent clearance (Fig. 4, C and D). Under oscillatory flow patterns in the presence of ADAMTS13, the VWF-platelet tangles and knots that formed on the endothelial surface bear resemblance to the pattern of platelet accumulation during the initiation of venous thrombosis in the stenosis model (*15*). Together, these data provide a potential explanation for why venous thrombi are initiated/develop specifically within the venous valve sinus or areas of aberrant flow rather than in other locations. Previously, Herbig and Diamond (*38*) reported the ability of soluble VWF to unravel at very high shear stress to form complex VWF fibers/tangles that exhibited resistance to ADAMTS13. This ADAMTS13 resistance is likely manifest in the same manner (associated with string tangling/knotting). However, these fibers only form in the absence of ADAMTS13 and at supraphysiological shear, which is in contrast to the low shear/multidirectional flow-induced tangling in our model. Similar low disturbed flow patterns have been reported upstream of the ligation point in the murine stenosis model of DVT, suggesting that there may be similar involvement of VWF tangle formation in this model (*39*).

The binding of platelets to VWF strings over the endothelial surface under venous flow alone does not represent thrombosis. This is due to the absence of platelet agonists such as collagen and thrombin as the release of second messengers that are necessary to drive further platelet activation. Although the GPIbα-VWF A1 domain interaction can transduce a mechanosensitive signal into the platelet resulting in repeated release of Ca^2+^ from intraplatelet stores (Fig. 2, D to F), this alone does not cause platelet activation (*16*, *33*). We previously reported that this signaling causes so-called “platelet priming,” characterized by activation of platelet surface α_IIb_β_3_, but with little evidence of degranulation required for normal hemostatic plug formation (*16*). Therefore, for these VWF-bound platelets to initiate thrombus formation, there is appreciable evidence that the subsequent platelet-dependent recruitment of leukocytes is required (*12*, *15*). Neutrophils are increasingly recognized as being relevant to DVT development (*12*). In the murine stenosis model, neutrophils are the first and most abundant leukocyte subtype recruited during thrombus initiation (*12*). The mechanisms responsible for specific VWF-bound platelet-dependent neutrophil recruitment have been debated. Platelet P-selectin appears to be unlikely because platelet binding to VWF under flow does not drive appreciable degranulation necessary for P-selectin exposure. However, P-selectin has the potential to recruit all leukocytes that express P-selectin glycoligand-1 (PSGL-1) and so is unable to confer such a high degree of neutrophil specificity (*40*). Consistent with this, platelet P-selectin appears to play only a minor role in the development of venous thrombosis in the murine stenosis model (*12*). This previously led us to hypothesize that an alternative means of VWF-bound platelet-dependent leukocyte recruitment must exist with the potential to exert a greater degree of neutrophil specificity (*16*, *18*).

We previously demonstrated that VWF-bound platelets can recruit neutrophils in a manner that is dependent on activated α_IIb_β_3_ that ensues from platelet priming (*16*). This interaction only occurs effectively at low venous shear and is unable to resist higher forces associated with arterial flow (Fig. 1). Using endothelial released VWF strings instead of immobilized VWF, we now show that VWF-platelet strings exhibit a high degree of specificity for recruiting neutrophils (Fig. 3, A and B, and movie S2). Neutrophil recruitment is in part dependent on activated α_IIb_β_3_ and its counter receptor, SLC44A2 (Fig. 3D). Neutrophil binding does not occur in the absence of platelets excluding the possibility that neutrophils bind directly to VWF. We also identified a role for P-selectin in neutrophil recruitment. On the basis of our previous findings, this is likely consistent with an auxiliary role for endothelial P-selectin in neutrophil recruitment/adherence to VWF strings on the endothelial surface (*16*), which corroborates the murine stenosis model data that report that endothelial P-selectin contributes to leukocyte recruitment (*12*). This, therefore, suggests that the VWF-bound platelets are responsible for capturing leukocytes, but that their retention is assisted by endothelial P-selectin, but endothelial P-selectin alone is insufficient to drive appreciable leukocyte recruitment, likely due to insufficient cell surface density of P-selectin. This may of course increase/change appreciably in conditions associated with more profound inflammatory states, such as infection. Moreover, once recruited, neutrophils underwent NET formation in a manner that was highly dependent on platelets, activated α_IIb_β_3_ and SLC44A2 (Fig. 3, E and F). Although P-selectin played a role in neutrophil recruitment, blocking P-selectin did not reduce the relative proportion of bound neutrophils that subsequently formed NETs (Fig. 3F). Together, these data are consistent with a model in which activated α_IIb_β_3_ on VWF-bound platelets recruits neutrophils in a manner that is aided by endothelial P-selectin, but that the signaling responsible for subsequent NET formation is more highly dependent on activated α_IIb_β_3_ and SLC44A2.

To explore this further, we developed a venous valve fluidic channel to mimic flow patterns that might exist in and around a venous valve of an individual with stasis-related risk factors (Fig. 6). A previous attempt using elastic valves corroborate some of our data, but the elasticated nature of the valves differs markedly from normal valve function (*41*). This channel enabled direct comparison of the formation of VWF strings/tangles within both the linear and pocket regions of the channel. Whereas the linear part of the channel was essentially devoid of VWF platelet strings through the protective effects of ADAMTS13, the low multidirectional flow within the pocket led to the formation of VWF tangles that recruited platelets. This process continued over time up to 30 min measured in this assay (Fig. 7C). It should be noted that these platelets did not build into occlusive aggregates such as those observed on a collagen surface but remained as small VWF-bound platelet foci (Fig. 7). These data strongly support the contention that aberrant flow within the valve pocket create an environment that enables the formation of ADAMTS13-resistant VWF tangles and provide an explanation for why the valve pocket represents the nidus for venous thrombosis initiation as opposed to the linear portions of veins. These VWF-platelet tangles promoted specific recruitment of neutrophils to the valve pocket, reinforcing the contribution of VWF, platelets, and low flow to the previously reported specific recruitment of neutrophils (rather than other leukocyte subtypes) during the early initiating phase of venous thrombosis (Fig. 8). The recruitment of neutrophils appears to involve contributions from activated α_IIb_β_3_ on platelets, SLC44A2 on neutrophils, and P-selectin on the endothelium.

How do these data inform our understanding of the initiation of DVT associated with stasis-related risk factors? We propose that the initiation and development of venous thrombosis might be separated into distinct phases (*42*). In phase 1, during the first phase, disturbed/aberrant flow within the valve pocket can promote the formation of VWF tangles capable of resisting ADAMTS13 proteolysis enabling the formation of VWF-platelet foci on the endothelial surface. This is primarily dependent on endothelial cell released VWF rather than the plasma pool. Therefore, inflammatory mediators (e.g., inflammatory cytokines, hypoxia, etc.) that may stimulate VWF release from endothelial cells within the venous valve sinus beyond the basal rate of VWF secretion would be predicted to augment this process. Because of the absence of thrombin or collagen at this early time point, these platelets are not activated but merely primed via the mechanosensitive signaling via GPIbα that leads to activation of α_IIb_β_3_ on the platelet surface. We might predict that this phase can potentially be quite easily reversed by restoration of “normal flow.” In some individuals, this may be simply restoring/improving movement to the lower limbs, which improves the irrigation of the inner leaflet of the valve to better facilitate proteolytic removal of the VWF tangles by ADAMTS13. In phase 2, the second phase, which occurs concomitantly, involves the preferential recruitment of neutrophils. This process relies on activated α_IIb_β_3_ on VWF-primed platelets and neutrophil SLC44A2 with an auxiliary role for endothelial P-selectin. Given that P-selectin does not provide leukocyte selectivity due to the presence of PSGL-1 on all leukocytes, we propose that the amount of P-selectin on the endothelial surface during these early phases does not, by itself, facilitate efficient leukocyte recruitment. Rather, the activated α_IIb_β_3_-SLC44A2 interaction provides the selectivity, which can be further stabilized via P-selectin–PSGL-1 interaction between the endothelium and neutrophils. The relevance of SLC44A2 in this process is corroborated by the protective effect of the rs2288904 SNP that encodes a R154Q substitution in the largest extracellular domain of SLC44A2 that disrupts binding to activated α_IIb_β_3_ (*16*, *20*). Individuals homozygous for *SLC44A2*(R154Q) have 30 to 50% reduced incidence of VTE (*20*, *21*). Moreover, mice deficient in SLC44A2 are protected against venous thrombosis in the stenosis model (*19*, *43*). Together, these and our data suggest that SLC44A2 contributes to neutrophil recruitment and downstream activation, leading to NET formation, representing the third phase of initiation. In phase 3, intravascular NET formation has been increasingly recognized as a prothrombotic driver in DVT (*3*, *12*–*14*, *44*). NETs can contribute to thrombus initiation via a multitude of proposed mechanisms including entrapment of blood cells, activation of platelets, and activation of the contact pathway of coagulation (*45*). In phase 4, this potentially provides a mechanism by which the first amounts of thrombin might be generated to enable platelet activation and fibrin deposition. These same processes may also enable the activation of monocytes that can provide a source of tissue factor to initiate the extrinsic pathway of coagulation. It is likely that these early procoagulant drivers are comparatively mild, meaning that this initiation phase may take several hours. It is therefore only at this point that the well-established procoagulant risk factors for VTE might influence thrombus development (factor V Leiden, prothrombin mutation, protein C/protein S deficiency, antithrombin deficiency, elevated factor VIII, etc.) (*3*). It is worth noting that these prothrombotic abnormalities can only increase the likelihood of thrombus development following the initiation phases 1 to 3. Once this initiation phase has begun, it is easier to understand how more classical prothrombotic mechanisms ensue involving platelet recruitment via plasma VWF to activated platelets and platelet activation driven by locally generated thrombin and second messenger release. Given the development of a nascent thrombus such as this over the surface of the intact endothelium, the local anticoagulant and fibrinolytic mechanisms likely cause the slow formation of the thrombus that may develop over many days or even weeks.

In summary, we provide a model for the initiation of venous thrombosis that explains the proclivity of DVT to develop in venous valve pockets. This involves a combination of aberrant flow that leads to VWF tangling that causes the formation of ADAMTS13-resistant VWF-platelet foci. These foci, in turn, recruit neutrophils via interactions that involve platelet α_IIb_β_3_, neutrophil SLC44A2 and endothelial P-selectin. Specific targeting of these mechanisms may provide major opportunities for improved thromboprophylaxis in patients at risk of VTE.

## MATERIALS AND METHODS

### Preparation of blood, platelets, leukocytes, and PFB

Blood was collected in either 3.13% citrate, 1× acid citrate dextrose (ACD) [85 mM trisodium citrate, 65 mM citric acid, and 111 mM d(+) glucose (pH 4.5)] (1.5/10 ml of blood), or 40 μM PPACK (Sigma-Aldrich). Informed consent was provided by all healthy volunteers before blood collection. The study was approved by the Imperial College Research Ethics Committee (approval reference 19IC5523). Blood was centrifuged for 10 min at 150*g* to separate platelet-rich plasma from RBCs and leukocytes. Platelet-rich plasma was supplemented with ACD (1:10), 75 mU of apyrase (Sigma-Aldrich), and 100 nM prostaglandin–E1 (PGE-1) (Sigma-Aldrich) and centrifuged at 1500*g* for 10 min. Platelets were washed twice with Hepes Tyrode (HT) buffer [137 mM NaCl, 2 mM KCl, 0.3 mM NaH_2_PO_4_, 1 mM MgCl_2_, 5.5 mM glucose, 5 mM Hepes, and 12 mM NaHCO_3_ (pH 7.35)] supplemented with 0.35% bovine serum albumin (HT-BSA) in the presence of apyrase/PGE-1. Washed platelets were resuspended in HT-BSA or recombined with washed RBCs and/or leukocytes as required. For RBC and leukocyte preparation, RBCs and leukocytes layers were resuspended/washed in HT-BSA. Cells were resuspended in HT-BSA and/or recombined with platelets to make PFB. For isolation of PBMCs and granulocytes, blood was collected in 3.13% citrate and prepared by Dextran-Percoll isolation.

### Platelet binding to VWF-coated channels

μ-slide VI Luer 0.1 or 0.4 channels (Ibidi) were coated with 2 μM VWF in phosphate-buffered saline (PBS) at 4°C overnight (*16*). Channels were washed with PBS and blocked with 2% BSA in PBS for 1 hour at room temperature. PFB containing 2.5 μM DiOC6 was perfused through VWF-coated channels for 3 min at different shear stress (1 to 45 dyne/cm^2^) using a Mirus Nanopump (Cellix) and platelet/leukocyte binding monitored by fluorescence microscopy using an Axio Vert.A1 Inverted Fluorescence Microscope (Zeiss). Channels were washed with HT-BSA, and platelet coverage across the channel was assessed by measuring mean fluorescence intensity across 10 fields of view through each channel.

### Platelet binding to VWF strings secreted from HUVECs

Pooled donor primary HUVECs (Lonza) were seeded in μ-slide I Luer 0.4 or 0.5 channels (Ibidi) coated with bovine fibronectin (50 μg/ml) and cultured in M199 (Sigma-Aldrich) supplemented with 20% fetal calf serum (FBS), 2 mM l-glutamine, penicillin (1 U/ml), streptomycin (0.1 mg/ml), endothelial cell growth supplement (30 μg/ml), and heparin (50 IU/ml; complete M199) at 37°C/5% CO_2_. To induce acute VWF release from HUVECs, cells were stimulated with 100 μM histamine/10 μM adrenaline in serum-free media for 5 min at 37°C under static conditions or were left unstimulated. Thereafter, PFB (containing 2.5 μM DiOC6) or washed platelets (containing 200 nM DiOC6) (±RBC, ±40 nM VWF, and ±150 nM anti–VWF A1 domain) were perfused through stimulated HUVEC channels at different shear stress (1 to 5 dyne/cm^2^) for 2 to 5 min using an ExiGo Pump (Cellix). Platelet binding was monitored using an Axio Vert.A1 Inverted Fluorescence Microscope (Zeiss). For quantification, at 2 min, the channel was rinsed with HT-BSA and VWF-platelet string length, and number was counted across 10 fields in each channel.

### Analysis of intraplatelet Ca^2+^ release

Isolated platelets were incubated with 1 μM Fluo-4AM (Invitrogen) for 30 min at 37°C. Platelets were washed in the presence of 75 mU of apyrase and 100 nM PGE-1, resuspended in HT-BSA, and used to make PFB containing 1 mM CaCl_2_ before initiation of the flow assay. HUVECs were stimulated with 100 μM histamine/10 μM adrenaline as before, PFB containing preloaded Fluo-4AM platelets was perfused through channels at 2.5 or 10 dyne/cm^2^, and the fluorescence of VWF-bound platelets was monitored for 3 min using an Axio Vert.A1 Inverted Fluorescence Microscope (Zeiss). The quantification of change in fluorescence of platelets was conducted using Slidebook 5.0 software. The fluorescence of individual platelets was followed for 40 s, with fluorescence measured every 150 ms. Baseline fluorescence was removed to detect Ca^2+^ spiking events. Maximum change in fluorescence (Δ Fluorescence) from baseline measurement was also determined for individual platelets.

### Leukocyte binding to VWF-platelet strings

To assess platelet-leukocyte interactions, PBMCs and granulocytes were isolated, washed, and resuspended in HT-BSA. Leukocytes were labeled with anti-CD16–allophycocyanin (APC), anti–CD14-APC, or anti–CD3-phycoerythrin antibodies (BioLegend) for 15 min to detect neutrophils, monocytes, or T cells, respectively. Confluent HUVECs in fluidic channels were stimulated with 100 μM histamine/10 μM adrenaline, as before, and PFB containing DiOC6-labeled platelets was perfused through the channel at 2.5 dyne/cm^2^ for 2 min to allow the formation of VWF-platelet strings. Thereafter, isolated/stained leukocytes were perfused at 0.5 dyne/cm^2^ for 6 min, and interactions between different leukocytes and VWF-bound platelets were monitored across a single field of view using an Axio Vert.A1 Inverted Fluorescence Microscope (Zeiss). Channels were washed with HT-BSA and leukocytes bound to VWF-platelet strings counted across the channel. To determine receptor dependencies for any platelet-neutrophil interactions, this assay was also performed in the presence of blocking antibodies against P-selectin (50 μg/ml), SLC44A2 (20 μg/ml), and β_2_-integrins (20 μg/ml) or 2 μM GR144053, which blocks α_IIb_β_3_, as previously described (*16*).

### Intraneutrophil Ca^2+^ signaling

To assess intracellular Ca^2+^ signaling in neutrophils following interaction with VWF-bound platelet strings, isolated granulocytes were loaded with 1 μM Fluo-4AM for 30 min at 37°C. Granulocytes were resuspended in HT-BSA containing anti–CD16-APC and 8 μM Hoechst for 15 min at room temperature. In parallel, platelets were incubated with 200 nM DiOC6 for 10 min, washed to removed free DiOC6, and perfused over stimulated HUVECs at 2.5 dyne/cm^2^ for 2 min to form VWF-platelet strings. Thereafter, Fluo-4AM-labeled neutrophils were perfused at 0.5 dyne/cm^2^. Neutrophils binding to VWF-platelet strings were identified, and images were captured every 150 ms to assess Ca^2+^-mediated changes in fluorescence using an Axio Vert.A1 Inverted Fluorescence Microscope (Zeiss).

### NET detection

To detect formation of NETs, staining for CitH3 was performed as previously described (*16*). HUVECs were cultured in channels. PFB was prepared as before, and granulocytes were isolated and labeled with anti–CD16-APC. Granulocytes were resuspended in M199. HUVECs were stimulated with 100 μM histamine/10 μM adrenaline for 5 min, followed by the perfusion of PFB for 1.5 min at 5 dyne/cm^2^ in the presence and absence of blocking antibodies against VWF A1 domain. PFB was washed out with M199, and granulocytes were perfused through channels at 0.5 dyne/cm^2^ for 10 min in the absence and presence of blocking antibodies against P-selectin (50 μg/ml), SLC44A2 (20 μg/ml), or β_2_-integrins (20 μg/ml) followed by wash out with M199 for 5 min to remove any unbound neutrophils from the channel. The assay was conducted at 37°C/5% CO_2_. The flow rate was then dropped to 0.001 dyne/cm^2^, and channels were left for 1 hour. At time points (0 to 1 hour) channels were fixed 4% paraformaldehyde/PBS for 15 min. Channels were washed with PBS twice and permeabilized with 0.5% Triton X-100 and 50 mM NH_4_Cl in PBS for 10 min. Following washout with PBS/0.1% Triton X-100, samples were then blocked with 3% BSA in PBS/0.1% Triton X-100 for 1 hour. A rabbit anti-CitH3 polyclonal antibody (10 μg/ml; Abcam, ab5103; lot GR273046-3) was incubated with the channels overnight at 4°C. Channels were washed and an Alexa-488–conjugated anti-rabbit immunoglobulin G antibody was added for 1 hour. Channels were then washed and mounted with Prolong Gold antifade mountant with 4′,6-diamidino-2-phenylindole (DAPI) before imaging. Images were taken using an Axio Vert.A1 Inverted Fluorescence Microscope (Zeiss) and an Eclipse Ti2 confocal microscope (Nikon).

### ADAMTS13 expression and quantification

Stably transfected human embryonic kidney 293 cells expressing full length ADAMTS13 were previously produced (*46*). Cells were cultured at 37°C/5% CO_2_. Upon reaching 70% confluency, cells were washed and cultured in OptiMEM. After 4 days, the conditioned medium was harvested and concentrated using a 50 kDa concentrator. ADAMTS13 concentration in conditioned media was measured using a human ADAMTS13 SimpleStep ELISA kit (Abcam) according to the manufacturer’s instructions.

### Influence of flow on ADAMTS13 clearance of VWF-platelet strings

Confluent HUVECs seeded in μ-slide VI Luer 0.4 channels were stimulated with 100 μM histamine/10 μM adrenaline as before. PFB prestained with 2.5 μM DiOC6 was perfused over HUVECs at 5 dyne/cm^2^ to form VWF-platelet strings. After 2 min, 50 nM ADAMTS13 in HT-BSA was perfused over the preformed VWF-platelet strings at different shear stresses (0.5 to 15 dyne/cm^2^) to monitor the shear dependency of ADAMTS13-dependent string clearance. Similar experiments were performed that included an additional 5 min period of oscillatory flow (±2.5 dyne/cm^2^) to induce VWF-platelet string tangling before addition of ADAMTS13. To assess the ability of VWF-platelet tangles to form in the presence of ADAMTS13, following HUVEC stimulation, PFB containing 5 nM ADAMTS13 was perfused through channels at ±2.5 dyne/cm^2^ under oscillatory flow. VWF-platelet string/tangle formation was monitored for 5 min using an Axio Vert.A1 Inverted Fluorescence Microscope (Zeiss).

### Influence of endothelial cell type, VWF, P-selectin, and α_IIb_β_3_ on VWF-platelet string and tangle formation

Confluent HUVECs, HMVEC (Lonza, cultured in Microvascular Endothelial Cell Growth Medium-2 BulletKit media containing 20% FBS), or HAEC (Lonza, cultured in Endothelial Cell Growth Medium-2 BulletKit media containing 10% FBS) seeded in μ-slide VI Luer 0.4 channels was stimulated with 100 μM histamine/10 μM adrenaline as before. PFB prestained with 200 nM DiOC6 was perfused over HUVECs at 5 dyne/cm^2^ to form VWF-platelet strings in the absence or presence of 2 μM GR144053, anti–P-selectin (50 μg/ml) or 200 nM anti–VWF A1 domain. After 2 min, a 2.5-min period of oscillatory flow (±5 dyne/cm^2^) to induce VWF-platelet string tangling was performed. VWF-platelet string/tangle formation was monitored for 5 min using an Axio Vert.A1 Inverted Fluorescence Microscope (Zeiss).

### Venous valve fluidic channels

To mimic the low/aberrant flow patterns that exist in individuals at risk of VTE associated with stasis-related risk factors, we designed a fluidic channel that incorporated a valve pocket. Channel dimensions are provided in fig S5. The channels consisted of a main channel through which PFB or blood could be drawn using a pump and a valve pocket with a small inlet to the pocket region, which was introduced to modulate flow patterns exclusively in the pocket (using multidirectional shear stress controlled by a separate pump) exclusively in the pocket. Molds were produced using a Polyjet 3D printer with the Objet Connex 3 in VeroClear with a glossy finish. Degassed polydimethylsiloxane (Dow Corning SYLGARD 184) was poured into molds and cured overnight at 70°C. The inlet and outlet holes were punched using a 2.5-mm biopsy punch, and the polydimethylsiloxane was bonded to a 22 mm–by–50 mm borosilicate glass coverslip using a plasma wand (Electro-Technic Products), followed by overnight incubation at 70°C. Holders were designed using Autodesk Inventor to allow fluid connection of valve channels and ensure channel stability. Channels were sterilized with 70% ethanol and coated with bovine fibronectin (150 μg/ml) in PBS for 1 hour. HUVECs were seeded into channels and left to adhere for at least 2 hours. Following cell adhesion, channels were attached to the pump tubing to enable flow of complete M199 media to produce an average shear stress of 5 dyne/cm^2^ within the linear region of the channel. To ensure accurate flow rates, measurements were taken with a flow meter (Sensirion). HUVECs were cultured within channels for 48 hours before experimentation. After 48 hours, channels were stained with sheep anti–VE-Cadherin (R&D Systems) and phalloidin (to stain F-actin) and mounted using ProLong Gold antifade mountant with DAPI. Images were taken using an Axio Vert.A1 Inverted Fluorescence Microscope (Zeiss) or by confocal microscopy (Nikon eclipse Ti2) to assess confluency.

### Flow assays in venous valve channels

To visualize the flow in the valve pockets and estimate shear stress distributions, computational fluid dynamic (CFD) simulations for the valve channels were conducted using OpenFOAM software with perfusion of a Newtonian fluid with a density of 1023 kg/m^3^ and a dynamic viscosity of 1.05 mPa·s to represent media supplemented with 20% FBS.

Channels seeded with HUVECs were connected to a LabV1 Intelligent Flow Rate Peristaltic Pump (Shenchen) for perfusion of media with a dampening system (*47*). Flow was initiated, and cells were cultured at 5 dyne/cm^2^ for 48 hours before experimentation. For flow experiments, the flow rate for LabV1 Intelligent Flow Rate Peristaltic Pump (Shenchen) connected to the channel outlet was set to create pulsatile flow up to 710 μl/min to induce a maximum shear stress of 5 dyne/cm^2^ within the linear section of the channel. For venous valve channels with the pocket inlet, the flow rate of the ExiGo pump (Cellix) was set to an oscillating flow rate with a maximum flow rate of 40 μl/min, a minimum flow rate of −10 μl/min, and a wave period of 3 s. The ExiGo pump was controlled by SmartFlow software (Cellix), allowed the control of specific flow rates and patterns. This generated multidirectional shear stress within the pocket region, without disturbing the unidirectional shear stress within the linear section of the channel. Blood was pulled over the channel via 0.8 mm in inner diameter and 10 cm in length inlet tubing connected to the channel inlet by male luer-to-barb fittings. The inner surface of the inlet tubing was blocked with 2% BSA/PBS for 30 min before use. For live imaging, an Axio Vert.A1 Inverted Fluorescence Microscope (Zeiss) was connected to an inverted charge-coupled device camera, and images were taken every 500 ms. The camera and microscope were operated by Slidebook 5.0 software.

### Valve channel—VWF-platelet binding flow assay

To assess formation of VWF-platelet strings/tangles within different regions of the valve channel, confluent HUVEC were stimulated with 100 μM histamine/10 μM adrenaline for 5 min before perfusion of whole blood anticoagulated with either 3.13% citrate or 40 μM PPACK and labeled with 2.5 μM DiOC6, and platelet binding was monitored. The peristaltic pump drawing blood from the channel outlet and a separate syringe pump inducing oscillatory flow at the valve pocket inlet were started to initiate perfusion through the channel. Platelet binding to VWF strings released by HUVECs was monitored in both the linear section and the middle of the valve pocket over 15 min using an Axio Vert.A1 Inverted Fluorescence Microscope (Zeiss). Subsequently, channels were washed out with M199, and platelet coverage was assessed across 10 fields of view within either the linear section or the middle of the valve pockets and quantified using the Slidebook 5.0 software.

### Valve channel—Neutrophil binding to VWF-bound platelets

Interactions between neutrophils and VWF-bound platelets in the linear and valve pocket sections of the valve channel were investigated as above, except that after 15 min, isolated granulocyte stained with anti–CD16-APC were perfused through the channels for 5 min and interactions between VWF-bound platelets and neutrophils were monitored. Valve channels were then washed out with M199, and the number of neutrophils bound to platelets and HUVECs was counted. To assess the contribution of platelets to neutrophil recruitment in the linear or pocket regions of the channel, the same assay was conducted in the absence of platelets. For this, either whole blood or platelet-free whole blood was perfused for 15 min before perfusion of neutrophils. After neutrophil perfusion for 5 min and washout, the number of neutrophils bound in the pocket or linear section of the channel was counted using an Axio Vert.A1 Inverted Fluorescence Microscope (Zeiss) over 10 fields of view and an average taken. This assay was also conducted in the presence or absence of an anti-SLC44A2 blocking antibody (20 μg/ml) to assess the influence of SLC44A2 to neutrophil binding to VWF-bound platelets. The number of neutrophils bound after perfusion was counted as described above. To account for any differences in platelet coverage, which may affect neutrophil binding across different fields of view, the number of bound neutrophils was normalized to the platelet coverage for each field of view. The evidence of NET formation was also explored 40 min after neutrophil perfusion through valve channels using DAPI to stain DNA.

### Statistical analysis

Statistical analyses were performed using Prism 10 software (GraphPad). Data comparisons were performed using one-way analysis of variance (ANOVA) with Tukey’s multiple post hoc comparisons or Dunnett’s multiple comparison test when comparing to a single variable. For comparison of two variables, unpaired two-tailed *t* test were performed. For data that was not normally distributed, nonparametric Kurskal-Wallis or two-tailed Mann-Whitney tests were performed. Statistical significance was considered at *P < 0.05, **P < 0.01, ***P < 0.001, and ****P < 0.0001. Information about the statistical tests is provided in the figure legends for the respective panels.
