# Supplementary material for: Role of von Willebrand factor (VWF), platelets, and aberrant flow in the initiation of venous thrombosis
Source: Sci Adv. 2025 Feb 5;11(6):eadr5250. doi: 10.1126/sciadv.adr5250 (PMC11797557; doi:10.1126/sciadv.adr5250)
Supplement: Supplementary file 1 — Figs. S1 to S6 Legends for movies S1 to S5 [file sciadv.adr5250_sm.pdf]

Science Advances
AAAS

# Supplementary Materials for

## Role of von Willebrand factor (VWF), platelets, and aberrant flow in the initiation of venous thrombosis

Laura J. Mereweather *et al.*

Corresponding author: James T. B. Crawley, j.crawley@imperial.ac.uk

*Sci. Adv.* **11**, eadr5250 (2025)
DOI: 10.1126/sciadv.adr5250

**The PDF file includes:**

Figs. S1 to S6
Legends for movies S1 to S5

**Other Supplementary Material for this manuscript includes the following:**

Movies S1 to S5

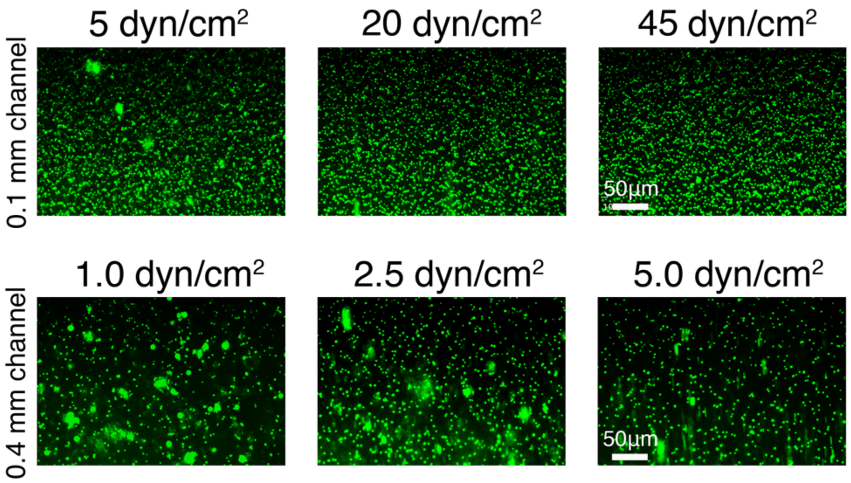


**Figure S1: Shear-dependence of platelet binding to immobilized VWF.** Representative images of platelet binding to VWF at different shear stress, following perfusion of 60 µl of PFB through a 0.1 mm height channel (upper panel) at 5, 20 or 45 dyn/cm$^2$. Platelet binding was comparable at 5, 20 and 45 dyn/cm$^2$ normalizing the volume of blood perfused over the VWF surface. Representative images of platelet binding to VWF at different shear stress, following perfusion of 400 µl of PFB through a 0.4 mm height channel (lower panel) at 1.0, 2.5 or 5.0 dyn/cm$^2$. Platelet binding was comparable at 1.0, 2.5 or 5.0 dyn/cm$^2$ normalizing the volume of blood perfused over the VWF surface. Scale bar represents 50 µm.

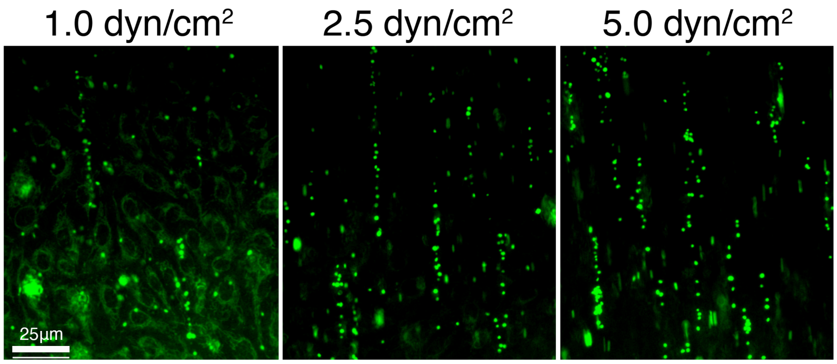


**Figure S2: Shear-dependence of platelet binding to VWF strings.** VWF-platelet strings after perfusion of PFB at 1.0, 2.5 or 5.0 dyn/cm$^2$ over HUVECs stimulated with 100 µM histamine/10 µM adrenaline for 5 mins. Representative images of VWF-platelet strings after perfusion of 320 µl of PFB. The number of VWF-platelet strings increased with shear even after controlling for the volume of PFB (i.e. number of platelets) perfused over the HUVEC surface. Left panel (1.0 dyn/cm$^2$) also presented in Fig 2A as a comparator. Scale bar represents 25 µm.

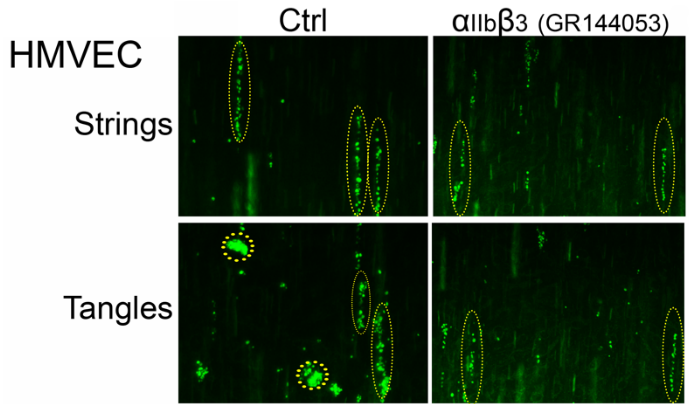


**Figure S3: VWF-platelet string and tangle formation on the surface of human microvascular endothelial cells (HMVEC).** Representative images of VWF-platelet strings through perfusion of DiOC6-labelled PFB under unidirectional flow at 5 dyn/cm$^2$ for 2 mins over confluent HMVECs stimulated with 100 μM histamine/10 μM adrenaline. PFB was perfused in the absence (Ctrl) or presence of GR144053 ($\alpha_{IIb}\beta_3$ blocker) domain (top panels). Thereafter, VWF-platelet string tangles were created in the same field of view by perfusing plasma-free blood forwards and backwards at ±5 dyn/cm$^2$ (± 1.2 l/min, wave period = 10 s) for 2.5 mins (bottom panels). Images are representative of n=3.

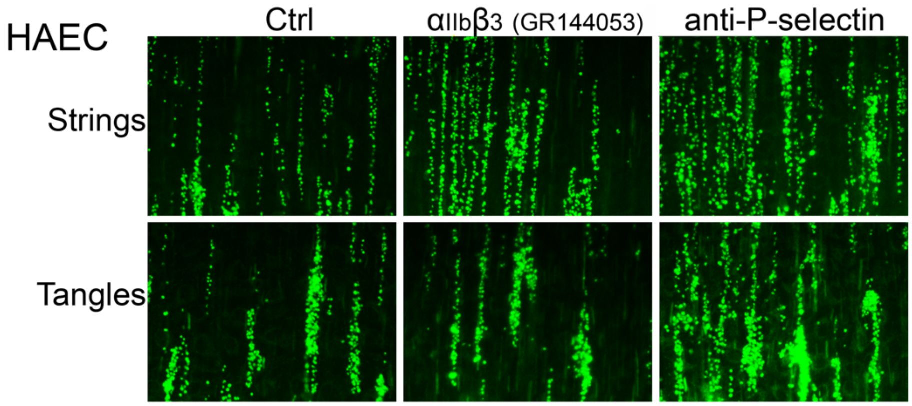


**Figure S4: VWF-platelet string and tangle formation on the surface of human aortic endothelial cells (HAEC).** Representative images of VWF-platelet strings through perfusion of DiOC6-labelled PFB under unidirectional flow at 5 dyn/cm$^2$ for 2 mins over confluent HAECs stimulated with 100 μM histamine/10 μM adrenaline. PFB was perfused in the absence (Ctrl) or presence of GR144053 ($\alpha_{IIb}\beta_3$ blocker) domain (top panels). Thereafter, VWF-platelet string tangles were created in the same field of view by perfusing plasma-free blood forwards and backwards at ±5 dyn/cm$^2$ (± 1.2 ml/min, wave period = 10 s) for 2.5 mins (bottom panels). Images are representative of n=3.

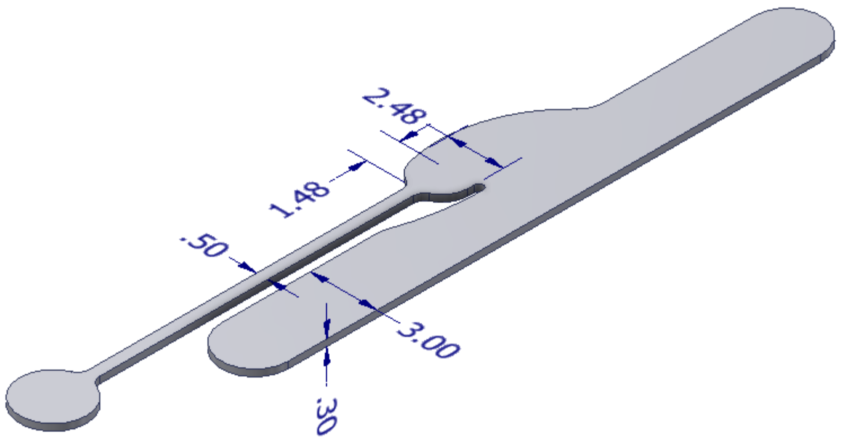


**Figure S5: Dimensions of the venous valve microfluidic channel.** The shape and dimensions of the venous valve fluidic channels. Channels consist of a linear section of width 3 mm, followed by expansion into a valve pocket of 2.48 mm and depth 1.48 mm, with a small channel of 0.5 mm width connected to the base of the valve pocket. Total channel length is 24 mm and height 0.3 mm.

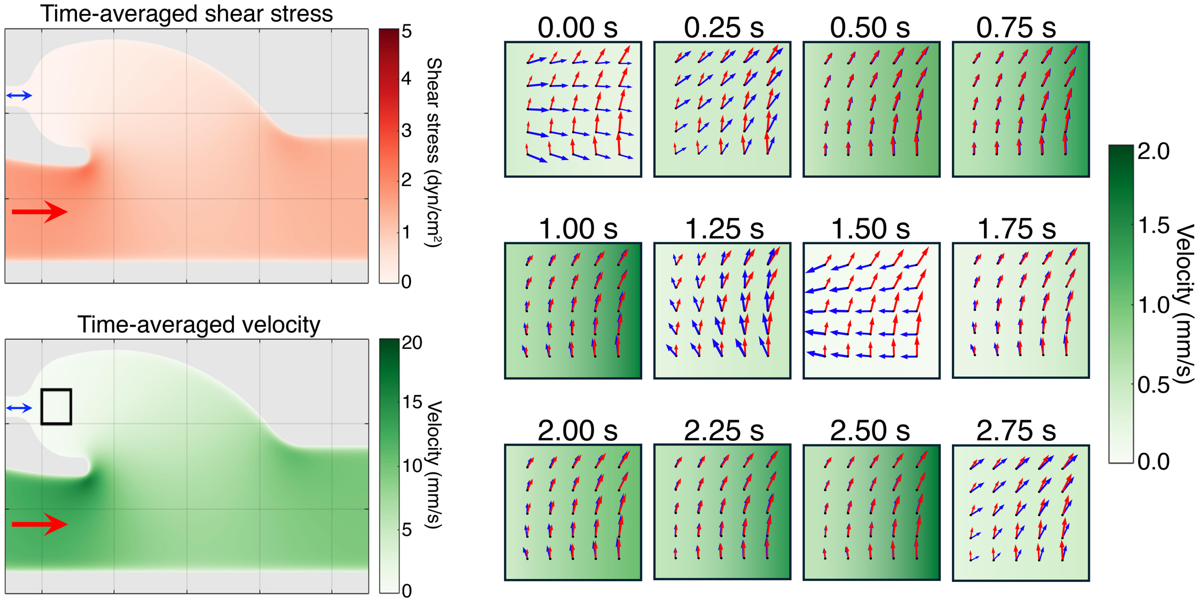


**Figure S6: CFD simulations in the venous valve channel.** CFD simulation of shear stress and changes to flow velocity in the pocket of venous valve channels. Upper left panel shows time averaged shear stress shows the average shear stress in the channel across the 3 s flow cycle. Lower left panel shows time averaged fluid velocity across the 3s flow cycle. The valve pocket region boxed is the region analyzed in the right panels. These present zoomed snapshots of velocity across the 3s cycle. Time averaged velocity vector is shown by the red arrows whereas the instantaneous velocity vector is shown by the blue arrows. Both shear stress and velocity remain low within the pocket although multidirectional flow is mediated through oscillations at the pocket inlet.

**Supplementary Movie Legends**

**Movie S1:** Intraplatelet $Ca^{2+}$ release within platelets bound to VWF strings at 10 dyn/cm$^2$. Platelets were pre-loaded with the $Ca^{2+}$-sensitive dye, Fluo-4 AM, and perfused over 100 μM histamine/10 μM adrenaline-stimulated HUVECs at 10 dyn/cm$^2$. Change in fluorescence of individual VWF-bound platelets was monitored over 40 s using a Zeiss inverted microscope at 40x, with images taken every 150 ms. Note repeated spiking in platelet fluorescence following binding to VWF strings under venous shear. Movie plays at 8x actual speed. Scale bar 25 μm.

**Movie S2: Binding of neutrophils to VWF-platelet strings.** Confluent HUVECs were stimulated with 100 μM histamine/10 μM adrenaline to induce VWF secretion. Washed platelets (labelled with DiOC6 – green) were perfused through the channel at 2.5 dyn/cm$^2$ for 2 mins to allow formation of VWF-platelet strings. Thereafter, isolated leukocytes containing anti-CD16 antibody to label neutrophils (red) were perfused at 0.5 dyn/cm$^2$ for 6 mins. Movie plays at 8x actual speed. Scale bar 25 μm.

**Movie S3: Tangling of VWF-platelet strings with oscillatory flow.** Following stimulation of HUVECs with 100 μM histamine/10 μM adrenaline, PFB was perfused at 2.5 dyn/cm$^2$ for 1 min at unidirectional flow. Thereafter, VWF-platelet string tangles were created by perfusing PFB forwards and backwards at ± 2.5 dyn/cm$^2$ (± 400 μl/min, wave period = 10 s) for 5 mins. Note how VWF strings tangle into knots. Movie plays at 8x actual speed. Scale bar 25 μm.

**Movie S4: Tangling of VWF-platelet strings with oscillatory flow in the presence of ADAMTS13.** Following stimulation of HUVECs with 100 μM histamine/10 μM adrenaline, PFB containing 5 nM ADAMTS13 was perfused forwards and backwards at ± 2.5 dyn/cm$^2$ (± 400 μl/min, wave period = 10 s) for 5 mins. Note how VWF strings tangled into compact knots are comparatively resistant to ADAMTS13 proteolysis, whereas extended strings are removed. Movie plays at 8x actual speed. Scale bar 50 μm.

**Movie S5: Formation of VWF-platelet tangles in the pocket of a venous valve microfluidic channel.** CFD representation of the time-averaged velocity of flow (top left) resulting from pulsatile flow through the main channel (red arrow) and low bidirectional flow through the valve inlet channel (blue arrow). Representation of the flow direction in the boxed area (lower left panels) displaying the contributions of the different flow channels to the flow direction in the valve pocket over a 3 s cycle. Confluent HUVECs cultured for 2 days under flow in valve channels were stimulated with 100 μM histamine/10 μM adrenaline for 5 mins. Subsequently, citrated whole blood labelled with DiOC6 to label platelets (green) was perfused through the channels. Movie shows VWF-platelet tangle formation between 10-12 mins, highlighting how multidirectionality impacts tangling of structures in the valve pocket using whole blood. Movie plays at 10x actual speed. Scale bar 50 μm.
